# Computational protocol to perform a spatiotemporal reconstruction of an epidemic

**DOI:** 10.1016/j.xpro.2023.102548

**Published:** 2023-09-16

**Authors:** Riccardo Nodari, Matteo Perini, Luca Fois, Lodovico Sterzi, Ester Luconi, Folco Vaglienti, Claudio Bandi, Elia Biganzoli, Massimo Galli, Francesco Comandatore

**Affiliations:** 1 Romeo ed Enrica Invernizzi Paediatric Research Centre, Department of Biomedical and Clinical Sciences, University of Milan, Milan, Italy; 2 Department of Environmental Health Sciences, Mailman School of Public Health, Columbia University, New York, NY, USA; 3 Department of Humanities, Section of Historical and Geographical Science, University of Pavia, Pavia, Italy; 4 Department of Biomedical and Clinical Sciences, University of Milan, Milan, Italy; 5 Department of Historical Studies, University of Milan, Milan, Italy; 6 Romeo ed Enrica Invernizzi Paediatric Research Centre, Department of Biosciences, University of Milan, Milan, Italy

**Keywords:** Bioinformatics, Health Sciences, Computer Sciences

## Abstract

Here, we present a computational protocol to perform a spatiotemporal reconstruction of an epidemic. We describe steps for using epidemiological data to depict how the epidemic changes over time and for employing clustering analysis to group geographical units that exhibit similar temporal epidemic progression. We then detail procedures for analyzing the temporal and spatial dynamics of the epidemic within each cluster. This protocol has been developed to be used on historical data but could also be applied to modern epidemiological data.

For complete details on the use and execution of this protocol, please refer to Galli et al. (2023).^1^

## Highlights

• Death-based clustering for epidemic reconstruction
• Protocol suitable for historical and modern epidemiological data analysis
• Spatial dynamics analysis of the epidemic on geographical maps

## Abstract (editor highlights)

Publisher’s note: Undertaking any experimental protocol requires adherence to local institutional guidelines for laboratory safety and ethics.

## Graphical abstract

**Figure:**
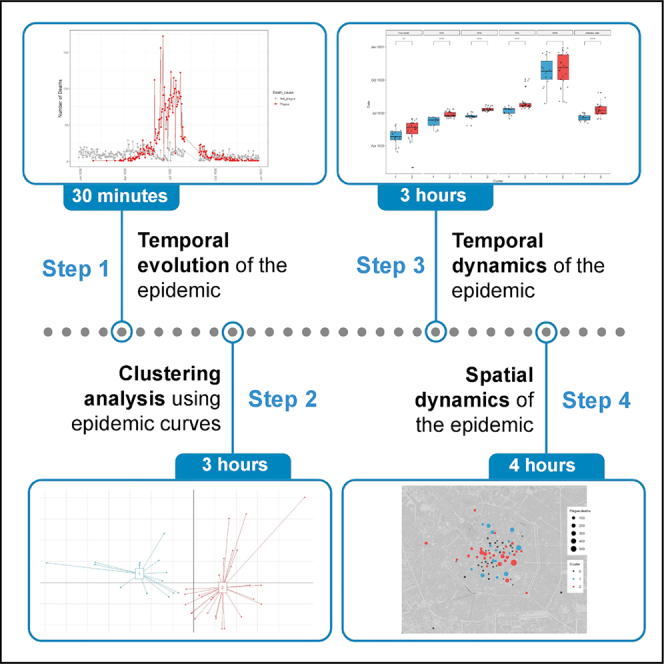

## Before you begin

The protocol below shows a way for the use of historical data (such as death records) to reconstruct the spatiotemporal evolution of a past epidemic. As an example, we analyzed the information contained in XVII century death registers of the city of Milan (Italy) to study the spatial and temporal diffusion of a plague epidemic inside the city.

This section includes the minimal software and hardware requirements, the installation procedures, as well as the format of the files to be processed throughout this protocol.

### Install R and RStudio

**Timing: 30 min**

1. R is a freely available language and environment for statistical computing and graphics.^2^ The latest version of R can be found at https://cran.r-project.org/ (Note that the protocol has been tested on R version 4.1.3 (2022-03-10)).
2. RStudio is a user-friendly integrated development environment for R.^3^ The installation of RStudio is not mandatory to follow the protocol. However, it makes viewing and interacting with files, packages, objects in the environment, tables, and graphs easy. RStudio can be found at https://www.rstudio.com/.

***Optional:*** QGIS is a free and open-source geographic information system^4^ that is used to create, edit, visualize, and analyze geospatial information. The latest version of QGIS can be found at https://www.qgis.org/en/site/.

### Install R packages needed for this protocol

**Timing: 10 min**

3. To run this protocol, it is required to previously install the R packages present in the “key resources table” as following:

> install.packages(“name_of_the_package”)

### Format your dataset or download our dataset from GitHub

**Timing: 10 min–1 h**

You can follow the protocol using your data or our sample dataset available on GitHub.

To follow the protocol step-by-step, you must have the data formatted in a dataset in which each row contains a single case. If you have a dataset with cumulative numbers, see below on how to format it properly for the protocol.

4. You can download our sample dataset from https://github.com/RiccardoND/STAR_protocol_epidemic_reconstruction (GitHub: https://doi.org/10.5281/zenodo.8214153) or directly clone the GitHub repository (∼10 Mb), by running the following command on your terminal.

> git clonehttps://github.com/RiccardoND/STAR_protocol_epidemic_reconstruction.

***Note:*** This repository includes all the data and code necessary to reproduce the protocol. In this dataset, each row contains the total number of “cases” (or deaths) for each cause of death, for each day (or other unit of time) and for each spatial location.

5. Format the table to obtain a single case in each row.

> df <- read.csv("TableS1.csv", na.strings = "NA")

> tab <- data.frame(lapply(df, rep, df$count))

> tab$count <- NULL

> tab$Date <- as.Date(tab$Date)

> tab <- tab[order(tab$Date),] #sort by date

***Note:*** It is always possible to format your dataset in other ways or add other information, but the protocol must be adjusted accordingly.

***Note:*** a version of the dataset already formatted as described in this step is available in the file TableS1_formatted.csv.

## Key resources table

**Table:** 

REAGENT or RESOURCE	SOURCE	IDENTIFIER
**Deposited data**		

Raw dataset about the 1630 plague epidemic data in Milan	Galli et al.^1^	https://github.com/RiccardoND/STAR_protocol_epidemic_reconstruction; https://doi.org/10.5281/zenodo.8214130
Step-by-step code	In this protocol	https://github.com/RiccardoND/STAR_protocol_epidemic_reconstruction; https://doi.org/10.5281/zenodo.8214153

**Software and algorithms**		

R 4.1.2	R Core Team^2^	https://www.R-project.org/; RRID: SCR_001905
RStudio (version 2022.07.1+554)	RStudio Team^3^	http://www.rstudio.com/; RRID: SCR_000432
QGIS 3.16.16	QGIS Geographic Information System^4^	http://www.qgis.org; RRID: SCR_018507
ggplot2 3.4.0 R package	Wickham^5^	https://cran.r-project.org/web/packages/ggplot2/index.html; RRID: SCR_014601
tidyverse 1.3.2 R package	Wickham et al.^6^	https://CRAN.R-project.org/package=tidyverse; RRID: SCR_019186
reshape2 1.4.4 R package	Wickham^7^	https://cran.r-project.org/web/packages/reshape2/index.html; RRID: SCR_022679
factoextra 1.0.7 R package	Kassambara and Mundt^8^	https://cran.r-project.org/web/packages/factoextra/index.html; RRID: SCR_016692
ade4 1.7-22 R package	Dray and Dufour^9^	https://cran.r-project.org/web/packages/ade4/
vegan 2.6-4 R package	Oksanen et al.^10^	https://cran.r-project.org/web/packages/vegan/
RColorBrewer 1.1-3 R package	Neuwirth^11^	https://cran.r-project.org/web/packages/RcolorBrewer/index.html; RRID: SCR_016697
inflection 1.3.6 R package	Christopoulos^12^	https://cran.r-project.org/web/packages/inflection/index.html
ggpubr 0.5.0 R package	Kassambra^13^	https://CRAN.R-project.org/package=ggpubr; RRID: SCR_021139
png 0.1-8 R package	Urbanek^14^	https://cran.r-project.org/web/packages/png/
vroom 1.6.3	Hester et al.^15^	https://cran.r-project.org/web/packages/vroom/index.html

**Other**		

Dell Precision 3630	Dell Technologies	https://www.dell.com/en-en/shop/cty/pdp/spd/precision-3630-workstation

## Step-by-step method details

### Tracing the temporal evolution of the epidemic

**Timing: 20–30 min**

To reconstruct the dynamics of an epidemic, it is helpful to start by plotting the number of deaths (or cases) that occurred at a specific time. In this case, we are going to build a time-series plot with two lines, one for deaths unrelated to the disease of interest, i.e., plague, and one for deaths related to plague. This step allows us to depict the temporal progression of the epidemic in the city and to detect any period with missing data or any other anomaly.

1. Prepare the workspace directory. Open RStudio and set the working directory path.

> setwd("yourpath")

2. Load the dataset of cases/deaths and related information and format it as shown in step 5 of “before you begin” section.

> head(tab)

**Table:** 

	Date	Death_cause	Parish
1	1630-01-01	Not_plague	S. Bartolomeo
2	1630-01-01	Not_plague	S. Donnino alla Mazza
3	1630-01-01	Not_plague	S. Martino in Nosiggia
4	1630-01-01	Not_plague	S. Nicolao
5	1630-01-01	Not_plague	S. Tommaso in Terramara
6	1630-01-02	Not_plague	S. Babila

**Figure 1:**
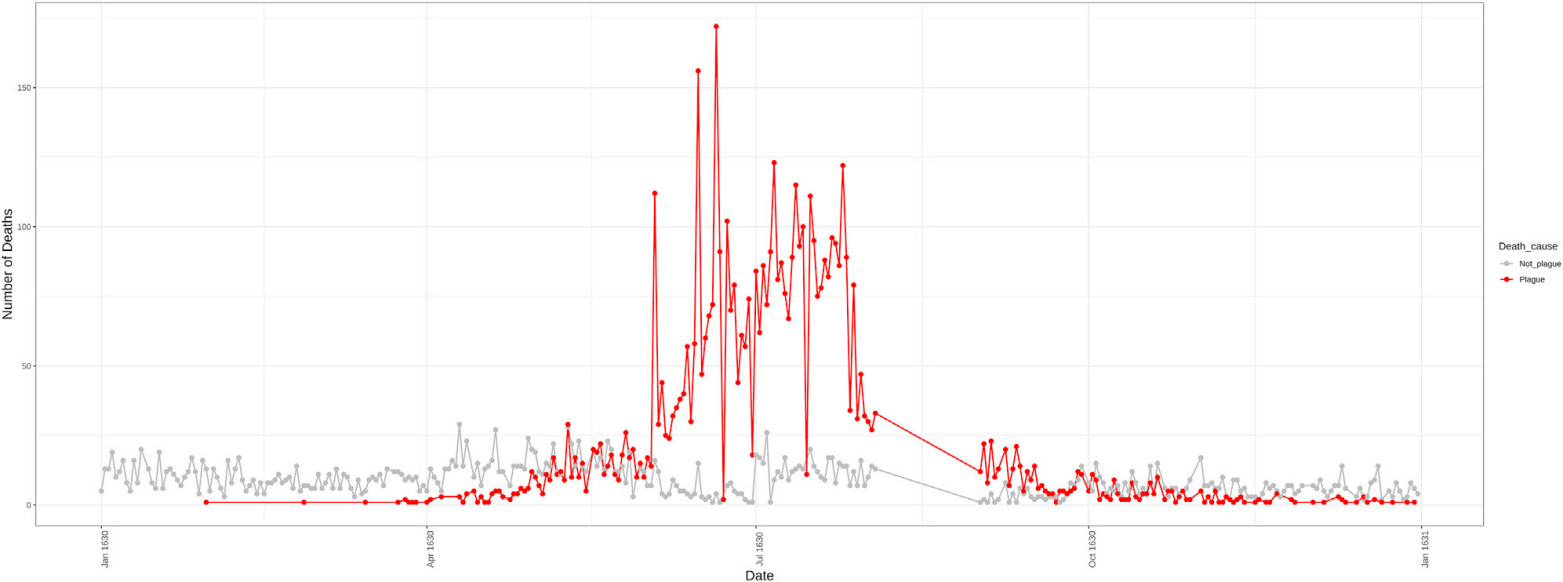
Daily number of deaths per cause of deaths The daily numbers of deaths are represented in separated colors depending on the cause of death: in red deaths related to plague, in gray deaths unrelated to plague.

3. Create a time-series plot that shows the progression of the daily number of deaths for each cause of death (in our case Plague or not plague) (Figure 1).

> library(ggplot2)

>library(tidyverse)

> tab %>%

 group_by(Date,Death_cause) %>%

 summarize(count = n()) %>%

 ggplot(aes(x = Date, y = count, col = Death_cause)) +

 geom_line(size = 0.7) +

 geom_point(size = 2) +

 theme_bw() +

 scale_color_manual(values=c("grey70","red")) +

 theme(axis.text.x = element_text(angle=90, size = 10),

  axis.title.x = element_text(size = 15),

  axis.title.y = element_text(size = 15)) +

 labs(y = 'Number of Deaths')

***Note:*** The plot compares the progression of deaths caused by the disease of interest to deaths related to any other cause: it could show the presence of any kind of seasonality, and the completeness of the dataset. The absence of a signal in the period between 4 and 30 August is due to reasons not related to the analysis.^1^

### Clustering of parishes on the basis of their epidemic curves

**Timing: 3–4 h**

In this step, we are going to group cases/deaths based on the progression of the epidemic. In particular, we are going to analyze the specific cumulative epidemiological curves of each geographical unit. In the example dataset about the 1630 plague epidemic in Milan, the geographical units are the parishes in which the city was divided and where the person died (i.e., territorial entities comparable to modern city neighborhoods). Other geographical units can be streets, houses, villages, districts, etc.

4. Load the R packages in the current RStudio session.

> library(tidyverse)

> library(reshape2)

> library(factoextra)

> library(ade4)

> library(vegan)

> library(RColorBrewer)

> library(inflection)

>library(ggpubr)

5. Load the table with one row per case, as shown in step 5 in the “before you begin” section (or skip if you have already loaded it in the previous step).
6. Build the cumulative curves of the plague deaths for each parish (or other geographical unit).
  a. filter for the cause of death or disease of interest.
    > tab_1630_peste <- droplevels(subset(tab, tab$Death_cause == "Plague"))
    > head(tab_1630_peste)

**Table:** 

	Date	Death_cause	Parish
334	1630-01-30	Plague	S. Maria alla Porta
567	1630-02-26	Plague	S. Marcellino
696	1630-03-15	Plague	S. Carpoforo
780	1630-03-24	Plague	S. Babila
801	1630-03-26	Plague	S. Bartolomeo
802	1630-03-26	Plague	S. Vincenzo in Prato
  b. build a matrix in which each column is a parish (or geographical unit), and each row is a day (or temporal unit).
    > t_1630_peste <- as.matrix(table(tab_1630_peste$Date, tab_1630_peste$Parish))
  c. Add to the matrix the days in which there are no recorded plague deaths.
    > days1630 <- seq(from=as.Date("1630-01-01"), to=as.Date("1630-12-31"), by=1)
    > absent_days <- as.Date(setdiff(days1630, as.Date(row.names(t_1630_peste))), origin="1970-01-01")
    > t_1630_peste_absent <- matrix(ncol=ncol(t_1630_peste), nrow=length(absent_days))
    > t_1630_peste_absent[is.na(t_1630_peste_absent)] <- 0
    > row.names(t_1630_peste_absent) <- as.character(absent_days)
    > t_1630_peste_all <- rbind(t_1630_peste, t_1630_peste_absent)
    ***Note:*** Select your temporal period of interest. In the example dataset, the cases span the year 1630, thus our range is from the 1st of January 1630 to the 31st of December of the same year.
  d. Order the table chronologically.
    > t_1630_peste_all_ord <- t_1630_peste_all[order(row.names(t_1630_peste_all)),]
  e. create a cumulative matrix.
    > peste_cum <- matrix(ncol=ncol(t_1630_peste_all_ord), nrow=0)
    > for (i in 2:nrow(t_1630_peste_all_ord)) {
     tmp <- colSums(t_1630_peste_all_ord[1:i,])
     peste_cum <- rbind(peste_cum, tmp)
    }
    > peste_cum <- rbind(t_1630_peste_all_ord[1,], peste_cum)
    > row.names(peste_cum) <-row.names(t_1630_peste_all_ord)
  f. Normalize the number of deaths by dividing the number of daily deaths in each parish by the total number of deaths in that parish (daily parish deaths/ total number of parish deaths in 1630).
    > peste_cum_norm <- apply(peste_cum, 2, function(x) x/max(x))
  g. Plot the cumulative relative frequency curves of the parishes plague deaths (Figure 2).
    > peste_cum_norm_m <- melt(peste_cum_norm)
    > colnames(peste_cum_norm_m) <- (c("Date", "Parish", "Count"))
    > peste_cum_norm_m$Date <- as.Date(peste_cum_norm_m$Date)
    > Cumulative_curves <- ggplot(peste_cum_norm_m, aes(x=Date, y=Count∗100, group = Parish)) +
     geom_line() +
     theme_bw() +
     ylab("Cumulative relative frequency of plague deaths (%)")
    ***Note:*** Some parishes may not have enough cases to build a reliable cumulative curve on the selected time range. We can choose a threshold under which the parishes will be excluded from the subsequent analyses. For example, we selected only the parishes with more than one plague death every two weeks during the epidemic.

**Figure 2:**
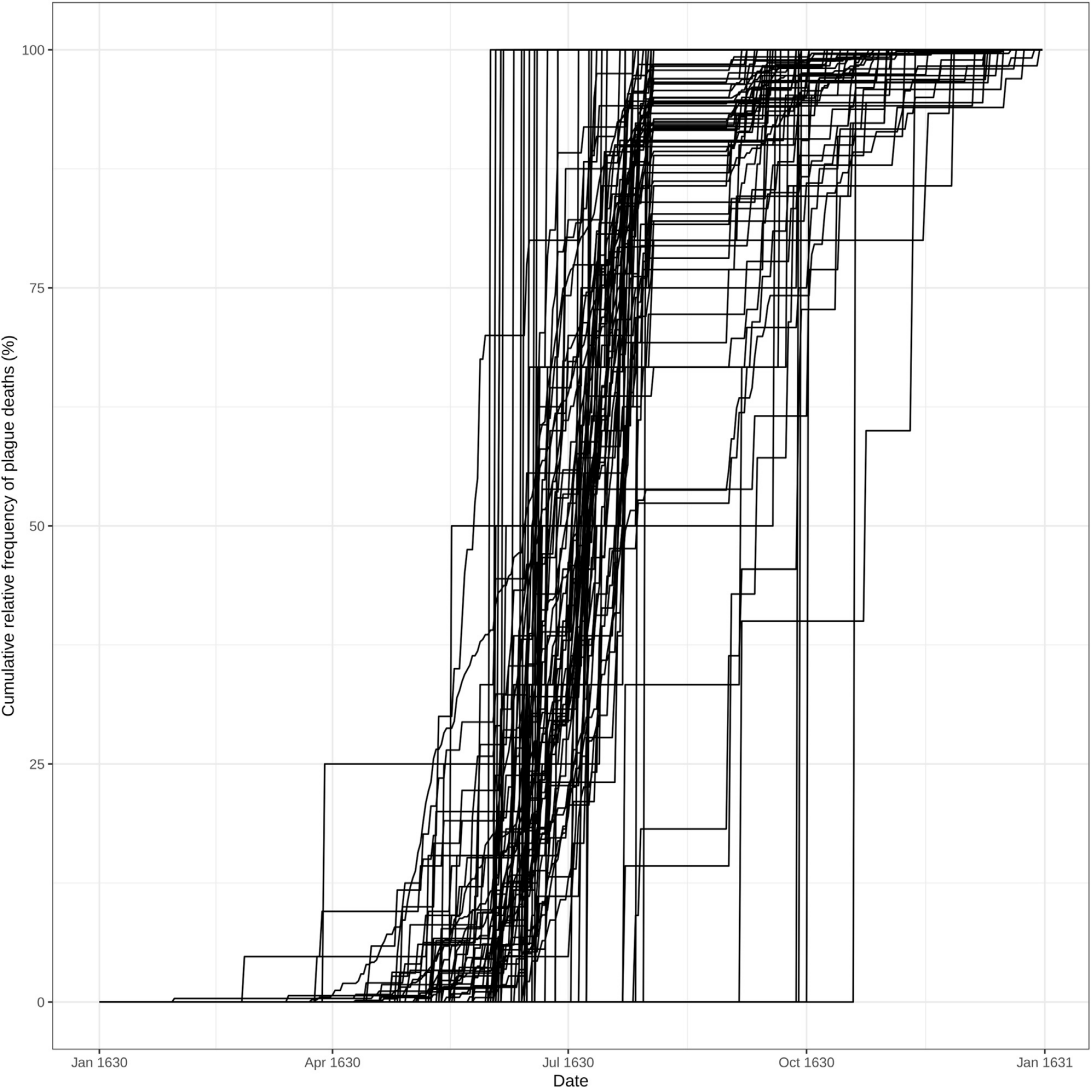
Cumulative relative frequency curves of plague deaths for each parish Each curve represents the cumulative frequency of plague deaths over time in each selected geographic unit (in this case, the parishes).
  h. Calculate the duration of the epidemic. To do so, we have to observe the epidemic curve produced in step 3 to manually determine the starting and ending period of the epidemic.
    ***Note:*** The epidemic period is not simply the time between the first and last case, in fact, as in our data, there may be some isolated cases before or after the epidemic. Thus, it is important to analyze the epidemic curve. In our data, the epidemic begins around the middle of March and continues throughout 1630.
  i. Calculate the death threshold. To select parishes with more than 1 death every two weeks, we have to look for parishes with more than 21 deaths (week of the epidemics / 2), as explained in Galli et al., 2023.^1^
    > weeks_of_epidemic <- 42
    > death_count_thr <- weeks_of_epidemic/2
    #21
  j. Remove parishes with fewer cases than the threshold and plot only the selected cumulative curves (Figure 3).
    > parr_sel <- peste_cum[nrow(peste_cum),] > death_count_thr
    > peste_cum_norm_sel <- peste_cum_norm[,parr_sel]
    > peste_cum_norm_melt <- melt(peste_cum_norm_sel)
    > colnames(peste_cum_norm_melt) <- c("Date", "Parish", "Count")
    > peste_cum_norm_melt$Date <- as.Date(peste_cum_norm_melt$Date)
    > cumulative_curves_sel <- ggplot(peste_cum_norm_melt, aes(x=Date, y=Count∗100, group = Parish)) +
     geom_line() +
     scale_color_manual(values = c("1" = "#1B9E77", "2" = "#D95F02")) +
     theme_bw() +
     ylab("Cumulative relative frequency of plague deaths (%)")

**Figure 3:**
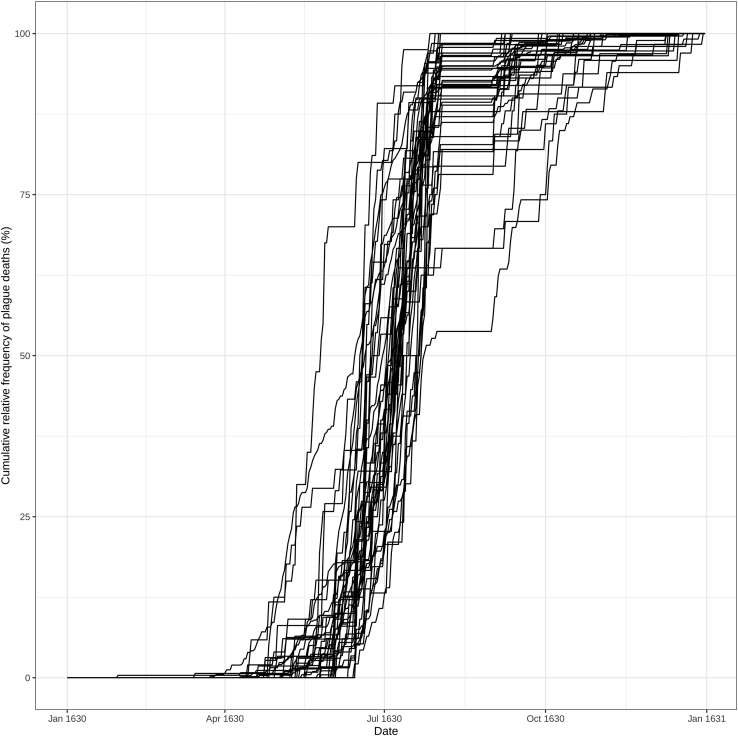
Cumulative relative frequency curves of plague deaths for each parish with at least 21 deaths
7. Perform the clustering analysis on the basis of the cumulative curves.
  ***Note:*** To cluster the parishes (or geographic units) in two or more groups, we are going to perform a k-means clustering on the result of the Principal Coordinates Analysis (PCoA) performed on the cumulative curves of plague deaths. In particular, we are going to compare the cumulative curves of the different parishes with each other to generate a distance matrix, which will be subjected to PCoA. Performing a PCoA on the dataset allows us to reduce the dimension of our data and to visualize them in two (or three) dimensions. Then we can apply k-means clustering, an unsupervised clustering algorithm that groups a dataset into a specific number of clusters (determined by silhouette analysis). The k-means algorithm will assign each observation (in our case each parish) to a cluster on the basis of their position on the PCoA space.^16^^,^^17^
  a. Calculate the Euclidean distance matrix between the cumulative curves of the different parishes.
    > dist <- dist(t(peste_cum_norm_sel))
  b. Perform the Principal Coordinates Analysis (PCoA) and make the scree plot (Figure 4).
    > pcoa <- cmdscale(dist, eig = T)
    > plot(pcoa$eig)
    > pcoa <- cmdscale(dist, 3)
    ***Note:*** First, we must determine the best number of axes for the PCoA analysis on the basis of the eigenvalues. The plot shows that the eigenvalues drastically drop down for the first three dimensions.

**Figure 4:**
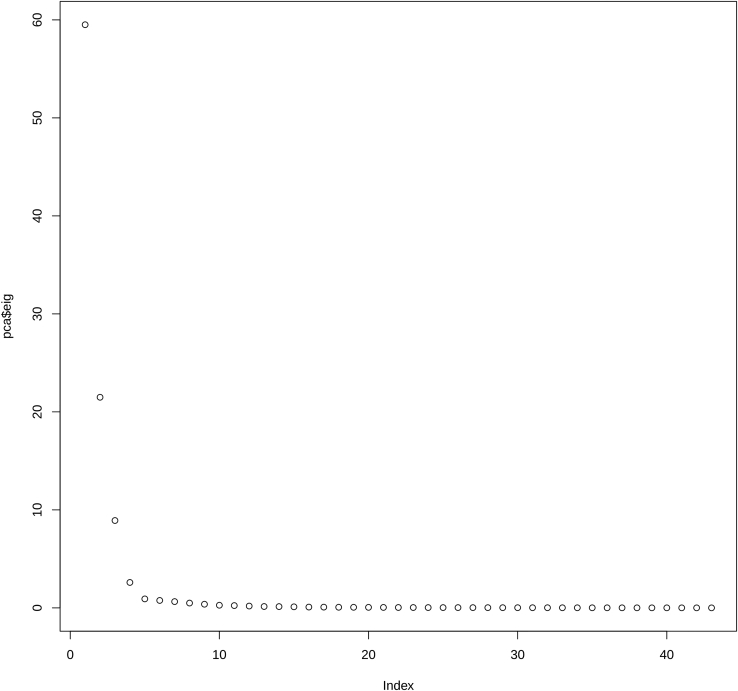
Eigenvalues of the Principal Coordinates Analysis (PCoA)
  c. Determine the optimal number of clusters in which the parishes (or geographic units) can be divided (Figure 5).
    > fviz_nbclust(pcoa, kmeans, method = "silhouette", k.max=10)
    ***Note:*** We determined the optimal number of clusters in the dataset using a popular cluster validation index: the average silhouette width method.^18^^,^^19^ In our example, the average silhouette width is maximized at the “number of clusters k” equal to two. Thus, this is the optimal number of clusters estimated by this method.

**Figure 5:**
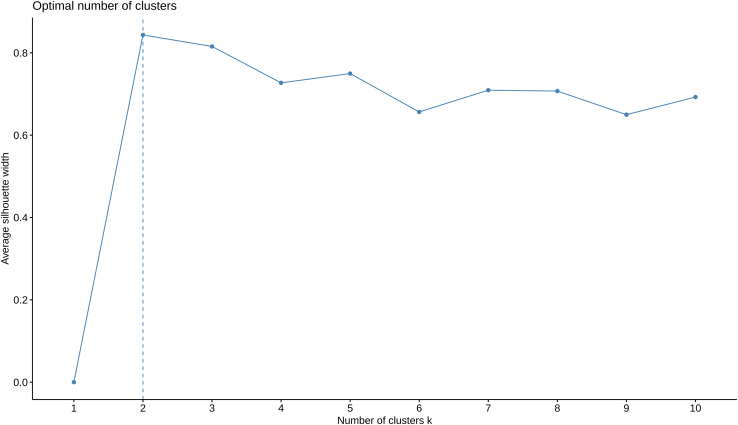
Optimal number of clusters defined by the silhouette method
  d. Clustering
    > f <- kmeans(pcoa, 2)
    > clusters <- as.matrix(f$cluster)
    > clus <- clusters[as.matrix(row.names(pcoa)),1]
8. Use the PERMANOVA test to determine if the separation between the clusters is statistically significant.

> a <- adonis2(pcoa ∼ clus, method='eu')

> p_value <- a$`Pr(>F)`[1]

> p_value

[1] 0.001

**Figure 6:**
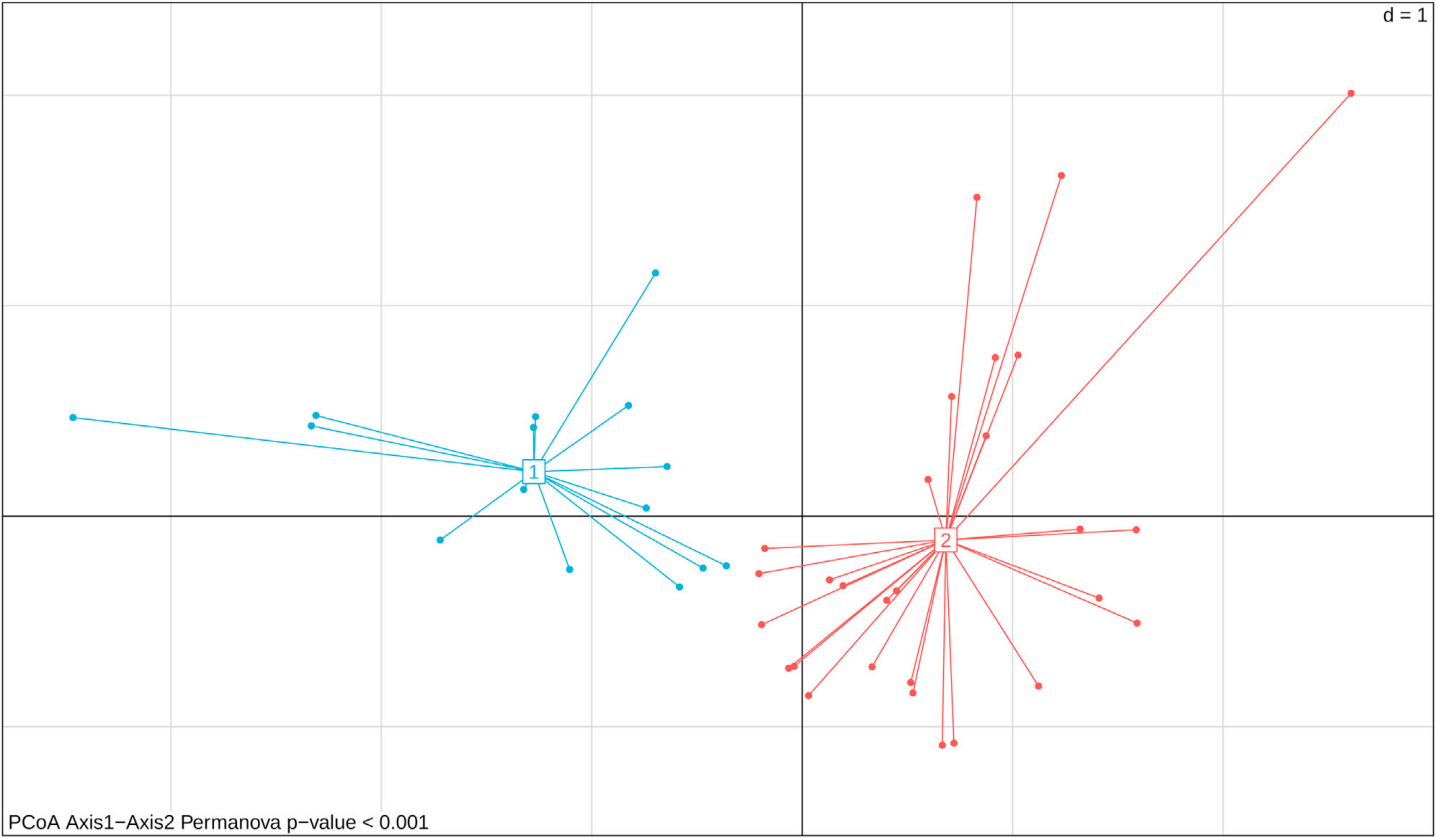
PCoA colored on the basis of the k-means clustering analysis PCoA using the Euclidean distance on the plague cumulative relative frequency curves. Each point represents a parish, and the colors represent the clusters: in blue parishes of cluster 1, in red parishes of cluster 2. The p value obtained from the PERMANOVA test is reported at the bottom-left of the figure.

9. Plot the results of the PCoA and the k-means clustering (Figure 6).

> plot_pcoa <- s.class(pcoa,

   as.factor(clus),

   col = c("#42a4cf","#ed4e4e"),

   cellipse = 0,

   sub = paste("PCoA Axis1-Axis2 Permanova p-value < ",

    p_value,

    sep = ""),

   xlim = c(-3.8, 3))

### Analyzing the temporal dynamics of the epidemic in each cluster

**Timing: 3–4 h**

The parishes, and therefore our cases, have been clustered on the basis of the temporal progression of the epidemic. Now we can analyze what are the differences and similarities between the clusters.

10. Load the necessary R packages in the current RStudio session.

> library(RColorBrewer)

> library(inflection)

> library(ggpubr)

**Figure 7:**
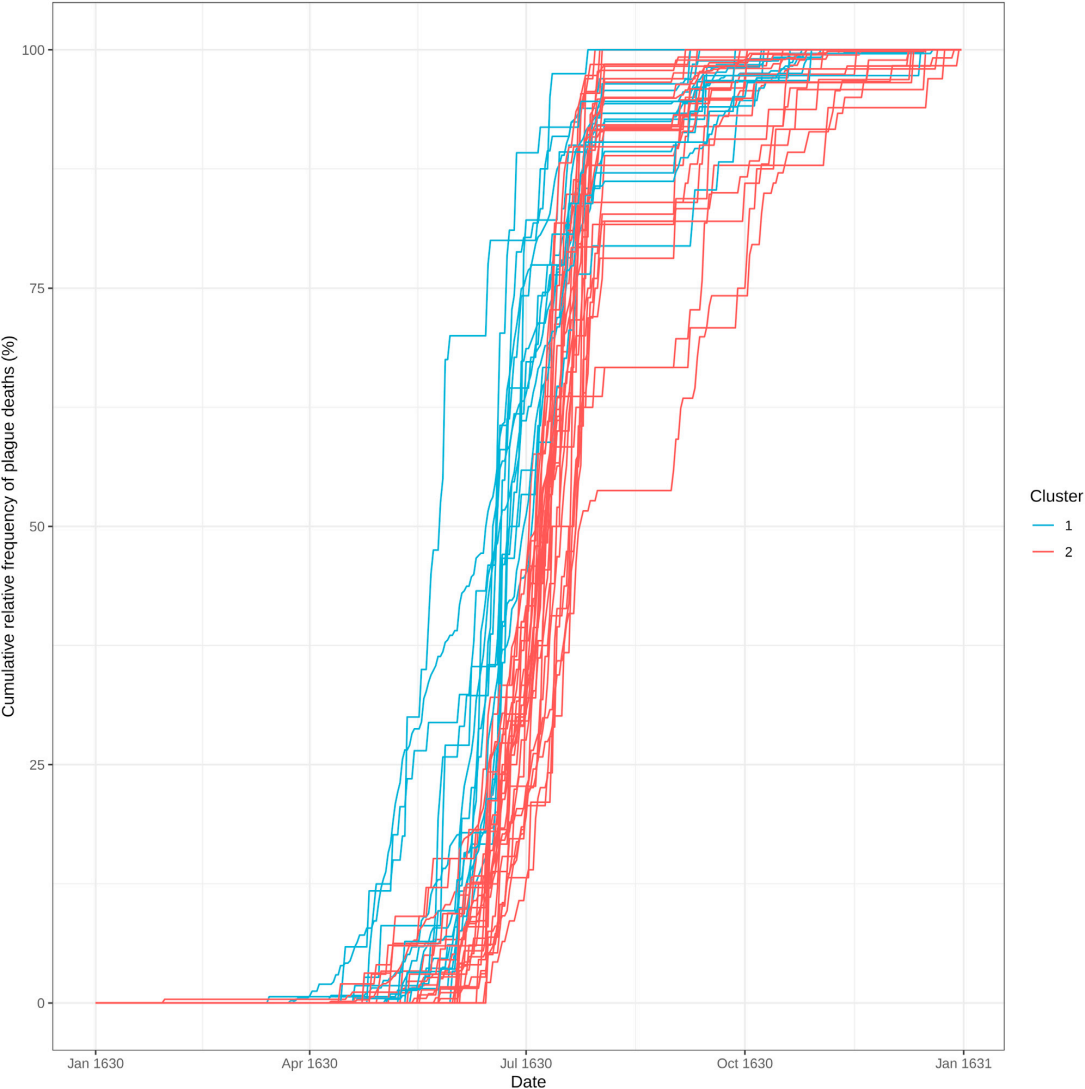
Cumulative relative frequency curves of plague deaths for each parish with at least 21 deaths colored by cluster

11. Color the cumulative relative frequency curves of plague deaths for each parish on the basis of their clusters (Figure 7).

> peste_cum_norm_melt <- melt(peste_cum_norm_sel)

> colnames(peste_cum_norm_melt) <- c("Date", "Parish", "Count")

> clusters <- as.matrix(f$cluster)

> peste_cum_norm_melt$Date <- as.Date(peste_cum_norm_melt$Date)

> peste_cum_norm_melt$Cluster <- as.character(clusters[as.matrix(peste_cum_norm_melt$Parish), 1])

> Cumulative_curves_sel_clusters <- ggplot(peste_cum_norm_melt, aes(x=Date, y=Count∗100, group = Parish, color = Cluster)) +

 geom_line() +

 scale_color_manual(values = c("1" = "#42a4cf", "2" = "#ed4e4e")) +

 theme_bw() +

 ylab("Cumulative relative frequency of plague deaths (%)")

12. Save a table with the information about the clusters and the corresponding parishes to be used later for further analysis on the clusters.

> palette <- c("#42a4cf", "#ed4e4e")

> tab_clusters <- data.frame(Parish = row.names(clusters), Cluster = > as.data.frame(clusters)$V1, Color = palette[as.matrix(clusters)])

> head(tab_clusters)

**Table:** 

	Parish	Cluster	Color
1	S. Andrea	2	#ed4e4e
2	S. Babila	1	#42a4cf
3	S. Bartolomeo	1	#42a4cf
4	S. Calimero	2	#ed4e4e
5	S. Carpoforo	1	#42a4cf
6	S. Donnino alla Mazza	1	#42a4cf

> write.csv(tab_clusters, file = "Clusters.csv", row.names = F)

13. Compare specific parameters relative to the epidemiological curves of the parishes of the two clusters.
  ***Note:*** As an example, we are going to determine: the first plague case for each parish, the inflection points of the cumulative curves, and the date at which the parishes of the two clusters reached 25%, 50%, 75%, and 100% of their total plague deaths.
  a. Calculate the dates on which the parishes of the two clusters reached the first plague death.
    > peste_cum_norm_melt_first <- peste_cum_norm_melt[peste_cum_norm_melt$Count > 0,]
    > peste_cum_norm_melt_first_nodup <- peste_cum_norm_melt_first[!duplicated(peste_cum_norm_melt_first$Parish),]
    > peste_cum_norm_melt_first_nodup[,3:4] <- NULL
    > colnames(peste_cum_norm_melt_first_nodup) <- c("Date_first_death","Parish")
  b. Calculate the dates on which the parishes of the two clusters reached 25% of total plague deaths.
    > peste_cum_norm_melt_25 <- peste_cum_norm_melt[peste_cum_norm_melt$Count >= 0.25,]
    > peste_cum_norm_melt_25_nodup <- peste_cum_norm_melt_25[!duplicated(peste_cum_norm_melt_25$Parish),]
    > peste_cum_norm_melt_25_nodup[,3:4] <- NULL
    > colnames(peste_cum_norm_melt_25_nodup) <- c("Date_25_death","Parish")
  c. Calculate the dates on which the parishes of the two clusters reached 50% of total plague deaths.
    > peste_cum_norm_melt_50 <- peste_cum_norm_melt[peste_cum_norm_melt$Count >= 0.5,]
    > peste_cum_norm_melt_50_nodup <- peste_cum_norm_melt_50[!duplicated(peste_cum_norm_melt_50$Parish),]
    > peste_cum_norm_melt_50_nodup[,3:4] <- NULL
    > colnames(peste_cum_norm_melt_50_nodup) <- c("Date_50_death","Parish")
  d. Calculate the dates on which the parishes of the two clusters reached 75% of total plague deaths.
    > peste_cum_norm_melt_75 <- peste_cum_norm_melt[peste_cum_norm_melt$Count >= 0.75,]
    > peste_cum_norm_melt_75_nodup <- peste_cum_norm_melt_75[!duplicated(peste_cum_norm_melt_75$Parish),]
    > peste_cum_norm_melt_75_nodup[,3:4] <- NULL
    > colnames(peste_cum_norm_melt_75_nodup) <- c("Date_75_death","Parish")
  e. Calculate the dates on which the parishes of the two clusters reached 100% of total plague deaths.
    > peste_cum_norm_melt_100 <- peste_cum_norm_melt[peste_cum_norm_melt$Count >= 1,]
    > peste_cum_norm_melt_100_nodup <- peste_cum_norm_melt_100[!duplicated(peste_cum_norm_melt_100$Parish),]
    > peste_cum_norm_melt_100_nodup[,3:4] <- NULL
    > colnames(peste_cum_norm_melt_100_nodup) <- c("Date_100_death","Parish")
  f. Calculate the dates on which the cumulative curves of the parishes of the two clusters change concavity, corresponding to the epidemic peak (also known as the inflection point).
    > infl_date_tab <- matrix(ncol=2, nrow=ncol(peste_cum_norm))
    > colnames(infl_date_tab) <- c("Inflection_date", "Parish")
    > for (i in 1:ncol(peste_cum_norm)){
     col = colnames(peste_cum_norm)[i]
     infl_date <- as.Date(bede(as.numeric(as.Date(row.names(peste_cum_norm))), peste_cum_norm[,as.matrix(col)],0)$iplast, origin = "1970-01-01")
     infl_date_tab[i, "Inflection_date"] <- as.character(infl_date)
     infl_date_tab[i, "Parish"] <- col
    }
  g. Merge all the data in one table.
    > all_tab_tmp <- merge(clusters, peste_cum_norm_melt_first_nodup, by.x="row.names", by.y="Parish")
    > colnames(all_tab_tmp)[1:2] <- c("Parish", "Cluster")
    > all_tab_tmp1 <- merge(all_tab_tmp, peste_cum_norm_melt_25_nodup, by="Parish")
    > all_tab_tmp2 <- merge(all_tab_tmp1, peste_cum_norm_melt_50_nodup, by="Parish")
    > all_tab_tmp3 <- merge(all_tab_tmp2, peste_cum_norm_melt_75_nodup, by="Parish")
    > all_tab_tmp4 <- merge(all_tab_tmp3, peste_cum_norm_melt_100_nodup, by="Parish")
    > all_tab <- merge(all_tab_tmp4, infl_date_tab, by="Parish")
    > head(all_tab)

**Table:** 

	Parish	Cluster	Date_first_death	Date_25_death	Date_50_death	Date_75_death	Date_100_death	Inflection_date
1	S. Andrea	2	1630-04-30	1630-06-23	1630-07-09	1630-07-20	1630-10-11	1630-07-20
2	S. Babila	1	1630-03-24	1630-05-10	1630-06-15	1630-06-30	1630-12-16	1630-06-21
3	S. Bartolomeo	1	1630-03-26	1630-06-07	1630-06-19	1630-07-12	1630-12-10	1630-06-11
4	S. Calimero	2	1630-04-20	1630-07-06	1630-07-21	1630-07-25	1630-11-19	1630-07-24
5	S. Carpoforo	1	1630-03-15	1630-06-18	1630-06-24	1630-07-14	1630-10-25	1630-06-21
6	S. Donnino alla Mazza	1	1630-05-31	1630-06-19	1630-06-27	1630-07-14	1630-10-09	1630-07-03
    > all_tab$Inflection_date <- as.Date(all_tab$Inflection_date )
    > all_tab2 <- melt(all_tab, id.vars = c("Parish", "Cluster"))
  h. Plot the results as boxplots and determine if the differences between the two clusters are statistically significant (Mann Whitney U test^20^ ) (Figure 8).
    > all_tab2$Cluster <- factor(all_tab2$Cluster, levels = c("1","2"), ordered = TRUE)
    > my_comparisons <- list(c("1","2"))
    > labels <- list("First death", "25%", "50%", "75%","100%", "Inflection date")
    #label name of facet
    > labels <- list("First_death" = "First death",
       "Date_25_death" = "25%",
       "Date_50_death" = "50%",
       "Date_75_death" = "75%",
       "Date_100_death" = "100%",
       "Inflection_date" = "Inflection date")
    > facet_labeller <- function(variable,value){
    return(labels[value])
    }
    > Clusters_boxplot <- ggboxplot(all_tab2, x = "Cluster", y = "value", fill = "Cluster") +
     scale_fill_manual(values=as.matrix(palette)) +
     geom_jitter(alpha=0.5, position = position_jitter(width = 0.3)) +
     facet_wrap(∼all_tab2$variable, ncol=6, labeller = facet_labeller ) +
     stat_compare_means(comparisons = my_comparisons, method = "wilcox.test", size=4, label = "p.signif", hide.ns = TRUE) +
     theme(legend.position = "none") +
     ylab( "Date") +
     theme(strip.text.x = element_text(size = 8))

**Figure 8:**
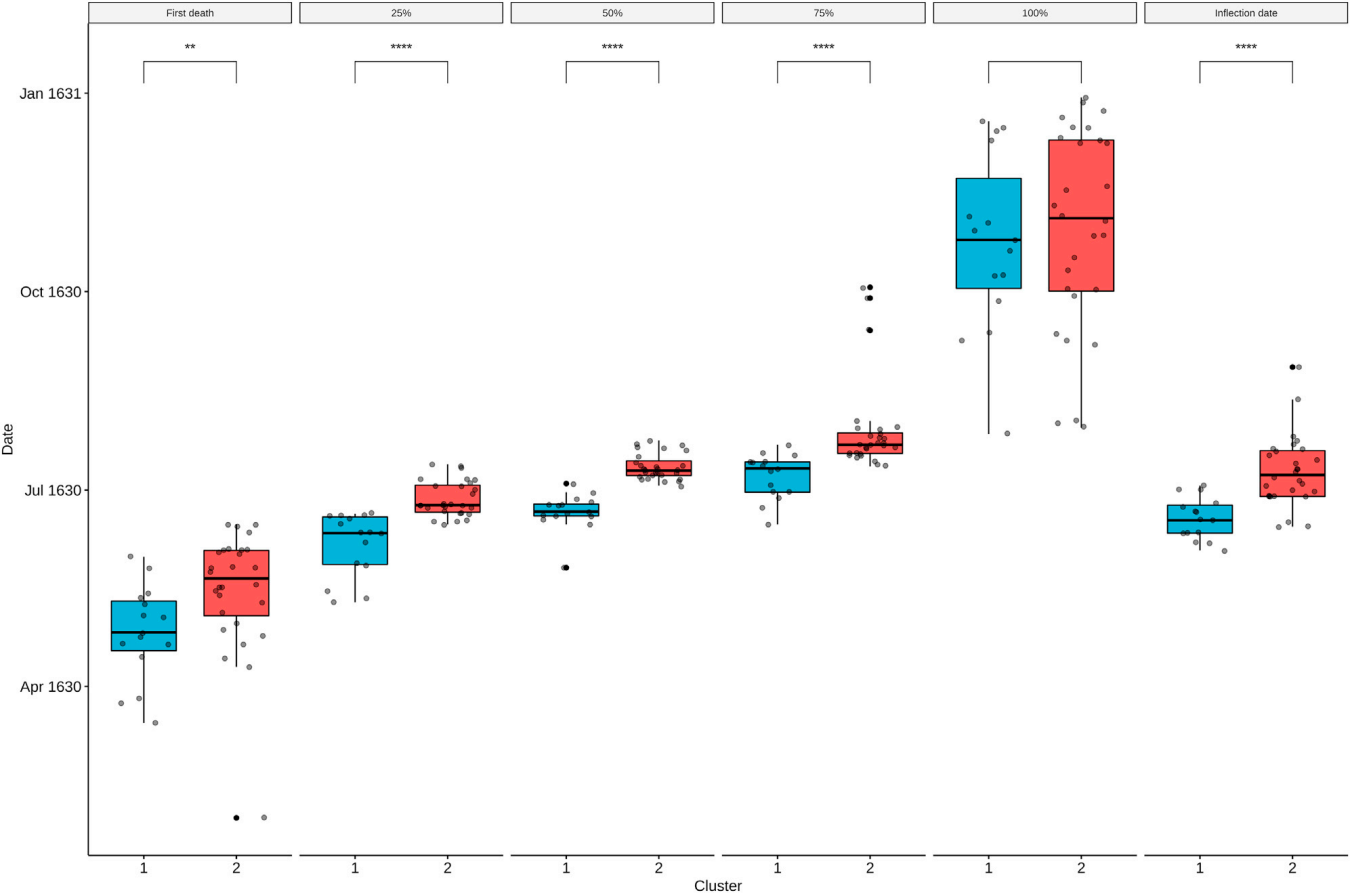
Comparison between the temporal progression of the epidemic in the two clusters From left to right, boxplots of the dates in which the parishes of the two clusters experienced the first plague death, 25% of total plague deaths, 50% of total plague deaths, 75% of total plague deaths, 100% of total plague deaths and the inflection point of the curves. In each boxplot, the dates for the two clusters were compared using a Mann-Whitney U test (‘∗’ p value < 0.05; ‘∗∗∗∗’ p value < 0.0001).
14. Visualize the epidemiological evolution of the epidemic in the parishes of the two clusters.
  a. Load the table with the epidemiological data, the table with the GPS information about the geographic units, in this case, the parishes, and the table with the clusters (see step 12).
    > df <- read.csv("TableS1.csv", na.strings = "NA")
    > gps <- read.csv("TableS2.csv", na.strings = "NA")
    > clusters <- read.csv("Clusters.csv")
  b. Produce a summary that integrates all the information for your dataset.
    > df2 <- data.frame(lapply(df, rep, df$count))
    > df3 <- df2 %>% select(-count)
    > tab <- left_join(df2, gps, by = "Parish")
    > tab$Date <- as.Date(tab$Date)
    > tab_cluster <- left_join(tab, clusters, by = "Parish")
    > tab_cluster_2 <- droplevels(subset(tab_cluster, !is.na(tab_cluster$Cluster)))
    #drop rows without cluster total number of cases in the 2 clusters = 7002
  c. Plot the weekly number of plague deaths for each cluster (Figure 9).
    > tab_cluster_2$Weeks <- as.numeric(format(tab_cluster_2$Date, "%W"))
    > tab_cluster_2$Cluster <- factor(tab_cluster_2$Cluster, levels=c(1,2))
    > tab_cluster_3 <- tab_cluster_2 %>% filter(Death_cause == "Plague")
    > cc_clusters <- tab_cluster_3 %>%
     group_by(Weeks,Cluster) %>%
     summarize(count = n()) %>%
     ggplot(aes(x = Weeks, y = count, col = Cluster)) +
     geom_line(size = 0.7) +
     geom_point(size = 2) +
     theme_bw() +
     scale_color_manual(values=c("#42a4cf","#ed4e4e")) +
     theme(axis.title.x = element_text(size = 12),
      axis.title.y = element_text(size = 12)) +
     labs(y = 'Number of Deaths')
    ***Note:*** The number of color values assigned to the function “scale_fill_manual” must be the same as the number of clusters.

**Figure 9:**
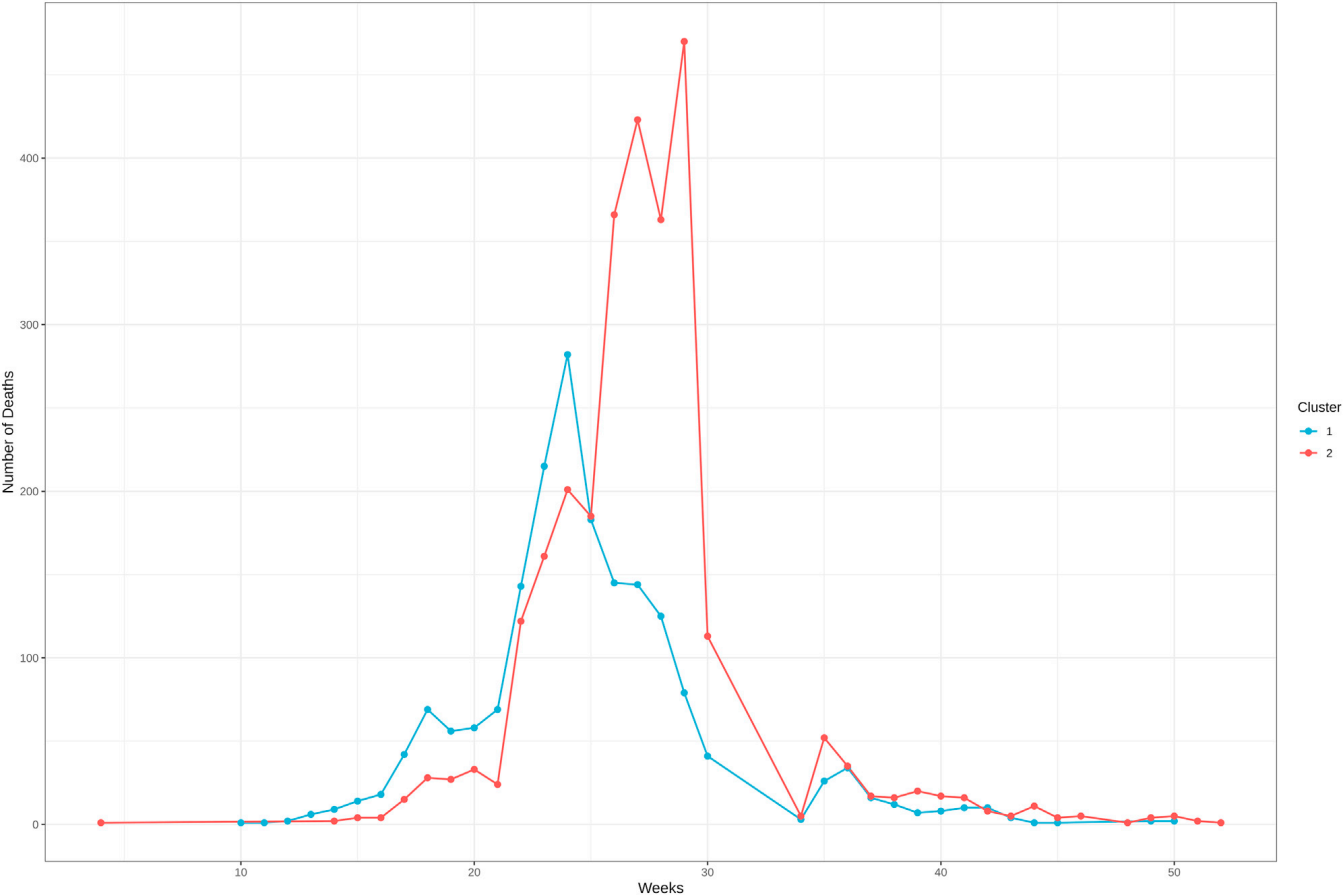
Weekly number of plague deaths for each cluster

### Analyzing the spatial dynamics of the epidemic in each cluster

**Timing: 4 h**

Generate a visualization of the distribution of the parishes of the different clusters on a map using the coordinates and the information about the parishes.

15. Load the R packages in the current RStudio session.

> library(png)

> library(grid)

16. Produce a summary table that integrates all the information for your dataset (see step 14b).

> head(tab_cluster)

# A tibble: 6 x 7

# Groups: Parish, Cluster, Color, Death_cause, Latitude [6]

**Table:** 

	Parish	Cluster	Color	Death_cause	Latitude	Longitude	Count
	<chr>	<dbl>	<chr>	<chr>	<dbl>	<dbl>	<int>
1	S. Alessandro in Zebedia	0	grey	Not_plague	9.19	45.5	9
2	S. Alessandro in Zebedia	0	grey	Plague	9.19	45.5	4
3	S. Ambrogio in Solariolo	0	grey	Not_plague	9.18	45.5	5
4	S. Ambrogio in Solariolo	0	grey	Plague	9.18	45.5	7
5	S. Andrea	2	#ed4e4e	Not_plague	9.20	45.5	23
6	S. Andrea	2	#ed4e4e	Plague	9.20	45.5	60

***Note:*** Latitude and Longitude are the columns used to indicate geographic coordinates.

17. Filter the dataset to remove all the parishes without geographic information.

> df <- tab_cluster %>% filter(!is.na(Latitude))

18. Create a new gray cluster (Cluster “0”) for the unassigned parishes (the parishes in this cluster are the one with less than 21 deaths, see step 6i for details).

> df$Cluster[is.na(df$Cluster)] <- 0

> df$Color[is.na(df$Color)] <- "gray"

19. Summarize the number of cases for each parish, cluster, death cause, and coordinates.

> df2 <- df %>% group_by(Parish, Cluster,Color, Death_cause, Latitude, Longitude) %>%

summarize(Count = n())

> head(df2)

# A tibble: 6 × 7

# Groups: Parish, Cluster, Color, Death_cause, Latitude [6]

**Table:** 

	Parish	Cluster	Color	Death_cause	Latitude	Longitude	Count
	<chr>	<dbl>	<chr>	<chr>	<dbl>	<dbl>	<int>
	S. Alessandro in Zebedia	0	grey	Not_plague	9.19	45.5	9
2	S. Alessandro in Zebedia	0	grey	Plague	9.19	45.5	4
3	S. Ambrogio in Solariolo	0	grey	Not_plague	9.18	45.5	5
4	S. Ambrogio in Solariolo	0	grey	Plague	9.18	45.5	7
5	S. Andrea	2	#D95F02	Not_plague	9.20	45.5	23
6	S. Andrea	2	#D95F02	Plague	9.20	45.5	60

20. Clean the table removing non-plague-related deaths.

> df2$Cluster <- factor(df2$Cluster, levels = c( "0","1","2"),ordered = TRUE)

> peste_clus_gps <- df2 %>% filter(Death_cause == "Plague")

> peste_clus_gps$Death_cause <- NULL

> peste_clus_gps <- peste_clus_gps[order(peste_clus_gps$Cluster),]

> peste_clus_gps$Latitude <- as.numeric(peste_clus_gps$Latitude)

> peste_clus_gps$Longitude <- as.numeric(peste_clus_gps$Longitude)

> peste_clus_gps$Count <- as.numeric(peste_clus_gps$Count)

21. Load your map stored as a *png* image and transform it into a raster image graphical object.

> map <- readPNG("positron_darker_2023.png")> map_2_plot <- rasterGrob(map, interpolate=TRUE)

22. Annotate the GPS coordinates of the four corners of the map image.

***Note:*** we can retrieve our base map image from QGIS by cropping the area of interest and annotating the GPS coordinates of the margins of our crop. This is essential to plot the points in their exact location on the map: the map itself needs to be referenced to the real GPS coordinates so that the point can be plotted using the real GPS coordinates available for each parish.

> gps_map <-data.frame(X = c(9.145413, 9.228967),

   Y = c(45.43978, 45.49275),

   fid = c(1,2),

   crop = c("BottomLeft", "TopRight"))

> xmin <- gps_map[gps_map$crop=="BottomLeft","X"] #Bottom Left margin

> ymin <- gps_map[gps_map$crop=="BottomLeft","Y"] #Bottom Right margin

> xmax <- gps_map[gps_map$crop=="TopRight","X"] #Top Right margin

> ymax <- gps_map[gps_map$crop=="TopRight","Y"] #Top Right margin

23. Find the aspect ratio of the png image and use the “zoom” variable to scale the output file size.

> img_width <- ncol(map)

> img_height <- nrow(map)

> aspect_ratio <- img_width/img_height

> zoom <- 15

***Note:*** With the aspect ratio of the map and a multiplicative factor (*zoom* variable), it is possible to save the final map image at different sizes maintaining good resolution. The reasonable value of the *zoom* variable depends greatly on the size (in pixels) and the shape of the initial crop of the map.

24. Plotting the parishes over the map according to the GPS coordinate. The size of the points is associated to the number of plague deaths and the color represents the cluster of the parishes.

***Note:*** The aspect ratio of the map must be maintained. To do so, we have to fix the width and height of the final plot file. RStudio may automatically plot the map with an incorrect aspect ratio. We strongly recommend saving the *png* file using the provided commands instead.

> p <-ggplot(peste_clus_gps, aes(Latitude,Longitude))+

 annotation_custom(map_2_plot, xmin=xmin, xmax=xmax, ymin=ymin, ymax=ymax)+

 geom_point(data=peste_clus_gps, aes( size = Count, color = Cluster))+

 scale_color_manual(values=c("grey30", "#42a4cf","#ed4e4e"))+

 labs(size="Plague deaths", color="Cluster") +

 xlim(xmin,xmax)+

 ylim(ymin,ymax)+

 theme_classic()+

 theme(axis.line=element_blank(),

  axis.text.x=element_blank(),

  axis.text.y=element_blank(),

  legend.text=element_text(size=zoom/2),

  legend.title=element_text(size=zoom/2),

  axis.ticks=element_blank(),

  axis.title.x=element_blank(),

  axis.title.y=element_blank(),

  panel.grid.major = element_blank(),

  panel.grid.minor = element_blank(),

  panel.background = element_blank(),

  legend.position = c(.92, .50) )

**Figure 10:**
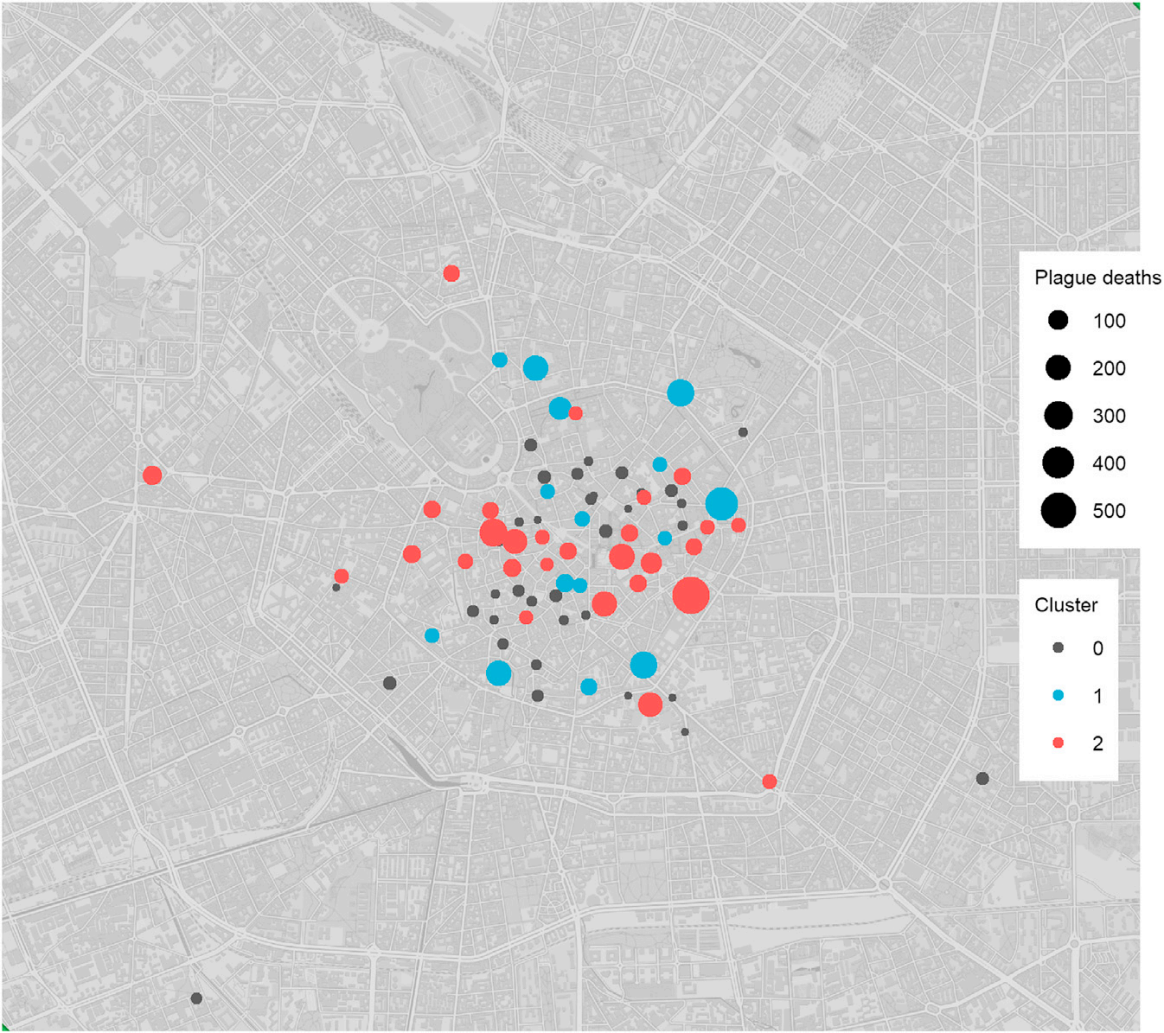
Geographical distribution of the parishes in the city of Milan Parishes localization on the map of the city of Milan. In blue, parishes of cluster 1; in red, those of cluster 2; in gray, parishes with less than 21 total plague deaths (not used in the clustering analysis). The size of the points represents the total number of deaths related to the plague experienced by the parish.

25. Save the final plot as *png* and scale it using the zoom variable (Figure 10).

> ggsave(p, filename = "Map_Clustering_plague_+21_deaths.png",

   device="png", units = "cm",

   width = aspect_ratio∗zoom,

   height = zoom)

Parishes localization on the map of the city of Milan. In blue, parishes of cluster 1; in red, those of cluster 2; in gray, parishes with less than 21 total plague deaths (not used in the clustering analysis). The size of the points represents the total number of deaths related to the plague experienced by the parish.

## Expected outcomes

This protocol has been designed to reconstruct the epidemiological dynamics of an epidemic using the recorded deaths and their geographical location.

The first outcome consists of a time-series plot where it is possible to compare the temporal evolution of the epidemics against the incidence of deaths unrelated to the disease of interest (Figure 1).

Then, the protocol performs a clustering analysis on the geographical units (i.e., parishes) on the basis of their cumulative relative frequency of plague deaths. The final outcome of this step is the Principal Coordinates Analysis (PCoA) plot, where the parishes have been colored on the basis of the clusters (Figure 6).

Once we find the clusters, we can start to analyze the temporal dynamics of the epidemic in each of them; in Figure 8, the boxplot and whiskers depict the different progression of the epidemics in the clusters.

Lastly, we analyze the spatial dynamics clusters by visualizing the geographical position of the parishes on a map: Figure 10 shows the position of the parishes over a historical map of the city of Milan. This map also shows the cluster of each parish and the number of plague-related deaths it experienced.

## Limitations

The clustering analysis used in this protocol does not rely on any kind of geographic information. Although this approach is advantageous when we are dealing with historical data for which geographic information is rarer or imprecise, this approach may be limiting for the analysis of datasets in which this information is available and reliable. In this case, the clustering analysis should consider the implementation of geographic information.

## Troubleshooting

### Problem 1

In step 5 of “before you begin” section, the data could be collected by more people also using different Operating Systems. In this case, it must be taken into account possible issues due to informatics compatibility, e.g., the use of different special characters to add a new line in a csv (comma-separated value) file.

### Potential solution

Open the csv file with a spreadsheet application (e.g., Office Excel or OpenOffice Calc).

In this application, we can easily apply any adjustment and export the spreadsheet as an xlsx or a csv file. The file should be correctly formatted and ready to be imported in R.

For csv files, we can import the table in R using the same lines of code in step 5 of “before you begin section”.

> df <- read.csv("TableS1.csv", na.strings = "NA")

For xlsx files (Excel format file):

> install.packages("readxl")

> library(readxl)

> df <- read_xlsx("TableS1.xlsx")

### Problem 2

At step 5 in the “before you begin” section, the table is too large to be loaded and handled in a reasonable time.

### Potential solution

When a table is too large (e.g., more than one million cells), the functions “read.csv” (for “csv files”) or “read.delim” (for tab delimited files) may take too much computational time and RAM to maintain the table in the environment. In this case, the “tibble” class can help reduce time and RAM needed to handle the dataset. Thus, to upload a large table it is better to use another R library such as “vroom”. The following code must be substituted to step 5 of the “before you begin” section:

> install.packages("vroom”)

> library(vroom)

> library(tidyverse)

> df <- vroom("TableS1.csv", delim = “,”)

> tab <- as_tibble(lapply(df, rep, df$count))

> tab$count <- NULL

> tab$Date <- as.Date(tab$Date)

> tab <- tab[order(tab$Date),] #sort by date

### Problem 3

At step 6, parishes name contains spelling errors.

### Potential solution

It is possible that the dataset contains spelling errors, or the same parish written in different ways (e.g., capital letters, spaces, etc.). In these cases, R does not consider them as the same parish. To find possible errors we can list all the parishes and manually check for errors.

> as.matrix(names(table(tab$Parish)))

Then we can correct them using R. As an example, consider a situation in which the parish of “S. Bartolomeo” is written in two different ways: “S. Bartolomeo” and “S. bartolomeo”.

> tab$Parish <- gsub("S. bartolomeo", "S. Bartolomeo", tab$Parish)

In this way we substituted all the cells containing "S. bartolomeo" with "S. Bartolomeo".

### Problem 4

At step 7c, the silhouette analysis indicates an optimal number of clusters higher than two.

### Potential solution

The protocol has been developed on the example dataset in which silhouette analysis finds that two clusters is the optimal number to classify the parishes. For this reason, this protocol can be directly applied only when the two clusters are found to be optimal.

Whenever the protocol finds that more than two clusters are needed to describe the dataset, the user should slightly modify the commands to be able to compare the clusters (e.g., in step 14c assign a color for each cluster to the function scale_fill_manual).

### Problem 5

In the section “analyzing the spatial dynamics of the epidemic in each cluster” (step 16), missing precise geographic location of geographical units.

### Potential solution

GPS information is not always available, particularly when dealing with old datasets that may refer to particular geographic locations (e.g., streets, parishes, neighborhoods) that changed names or disappeared over time.

The clustering approach applied in this protocol does not rely on any type of geographical information. Thus, you can follow the protocol from step 1 till step 14 without any specific geographic information (corresponding to the sections: “tracing the temporal evolution of the epidemic”, “clustering of parishes on the basis of their epidemic curves”, and “analyzing the temporal dynamics of the epidemic in each cluster”). Conversely, it is not possible to continue with the section “analyzing the spatial dynamics of the epidemic in each cluster” (from step 15) when GPS coordinate information is missing.

In the presence of approximate GPS coordinate information for the geographical units, a possible solution can be to group the geographical units into larger ones. For example, if you have only approximate GPS information about the parishes but you have precise information about the district in which the parishes are located, you can group all the parishes in their respective district creating larger geographical units. In this case, you have to format the dataset to conform to the larger geographical unit in the “before you begin” section (e.g., aggregating the deaths of the parishes of each district). Then, the newly formatted dataset can be used to perform the protocol at the district level from step 1 to the end.

## Resource availability

### Lead contact

Further information and requests for resources and reagents should be directed to and will be fulfilled by the lead contact, Riccardo Nodari (riccardo.nodari@unimi.it).

### Materials availability

This study did not generate new unique reagents.

## Data Availability

The accession number for the data and code reported in this paper is GitHub: https://doi.org/10.5281/zenodo.8214153.

## References

[1] Galli M., Nodari R., Perini M., Luconi E., Fois L., Vaglienti F., Bandi C., Biganzoli E., Comandatore F. (2023). A spatiotemporal reconstruction of the 1630 plague epidemic in Milan. iScience.

[2] R Core Team (2021). https://www.R-project.org/.

[3] RStudio Team (2020).

[4] QGIS Development Team (2022). QGIS Geographic Information System. http://www.qgis.org.

[5] Wickham H. (2009).

[6] Wickham H., Averick M., Bryan J., Chang W., McGowan L.D., François R., Grolemund G., Hayes A., Henry L., Hester J. (2019). Welcome to the Tidyverse. J. Open Source Softw..

[7] Wickham H. (2007). Reshaping Data with the reshape Package. J. Stat. Software.

[8] Kassambara A., Mundt F. (2017).

[9] Dray S., Dufour A.-B. (2007). The ade4 Package: Implementing the Duality Diagram for Ecologists. J. Stat. Software.

[10] Oksanen J., Blanchet F.G., Kindt R., Legendre P., Minchin P.R., O’hara R.B., Simpson G.L., Solymos P., Stevens M.H.H., Wagner H. (2013).

[11] Neuwirth E. (2014).

[12] Christopoulos D.T., Christopoulos M.D.T., Christopoulos D.T. (2022).

[13] Kassambara A. (2020).

[14] Urbanek S. (2013). R Package Version 0.1-7.

[15] Hester L., Wickham H., Bryan J. (2021). Vroom: Read and Write Rectangular Text Data Quickly. https://CRAN.R-project.org/package=vroom.R.package.version.1.

[16] Hartigan J.A., Wong M.A. (1979). A K-Means Clustering Algorithm. J. Roy. Stat. Soc. C Appl. Stat..

[17] James G., Witten D., Hastie T., Tibshirani R. (2013).

[18] Batool F., Hennig C. (2021). Clustering with the Average Silhouette Width. Comput. Stat. Data Anal..

[19] Rousseeuw P.J. (1987). Silhouettes: A graphical aid to the interpretation and validation of cluster analysis. J. Comput. Appl. Math..

[20] Nachar N. (2008). The Mann-Whitney U: A test for assessing whether two independent samples come from the same distribution. Tutor. Quant. Methods Psychol.

